# Supplementary material for: Engineering interfacial sulfur migration in transition-metal sulfide enables low overpotential for durable hydrogen evolution in seawater
Source: Nat Commun. 2024 Jul 22;15:6154. doi: 10.1038/s41467-024-50535-2 (PMC11263604; doi:10.1038/s41467-024-50535-2)
Supplement: Supplementary file 1 — supplementary information [file 41467_2024_50535_MOESM1_ESM.pdf]

Engineering interfacial sulfur migration in transition-metal sulfide enables low overpotential for durable hydrogen evolution in seawater

Table of Contents

Supplementary Figure 1. 2
Supplementary Figure 2. 3
Supplementary Figure 3. 4
Supplementary Figure 4. 5
Supplementary Figure 5. 6
Supplementary Figure 6. 7
Supplementary Figure 7. 8
Supplementary Figure 8. 9
Supplementary Figure 9. 12
Supplementary Figure 10. 13
Supplementary Figure 11. 14
Supplementary Figure 12. 15
Supplementary Figure 13. 16
Supplementary Figure 14. 17
Supplementary Figure 15. 19
Supplementary Figure 16. 20
Supplementary Figure 17. 21
Supplementary Figure 18. 22
Supplementary Figure 19. 23
Supplementary Figure 20. 24
Supplementary Figure 21. 25
Supplementary Figure 22. 26
Supplementary Figure 23. 27
Supplementary Figure 24. 28
Supplementary Figure 25. 29
Supplementary Figure 26. 30
Supplementary Table 1. 10
Supplementary Table 2. 11
Supplementary Table 3. 18

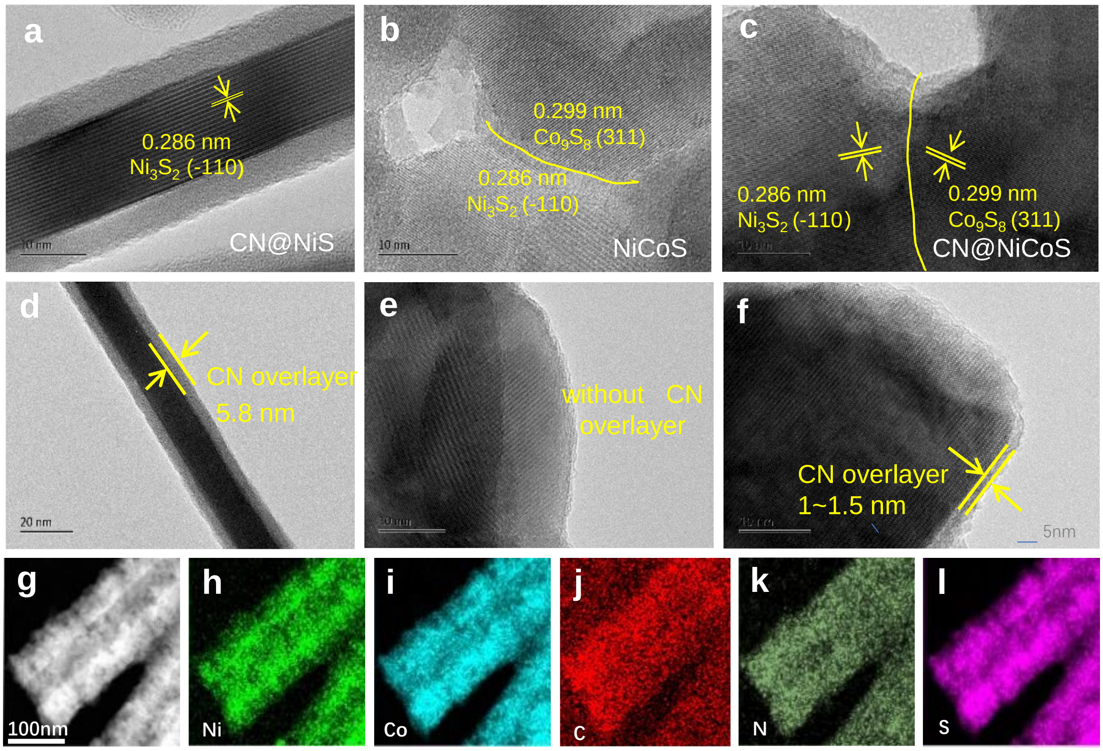

Supplementary Figure 1. Morphology of metal-sulfides catalysts with different structure and composition. TEM images (a,d) CN@NiS. (b,e) NiCoS. (c,f) CN@NiCoS. (g-l) HAADF-STEM image and corresponding element mapping of CN@NiCoS.

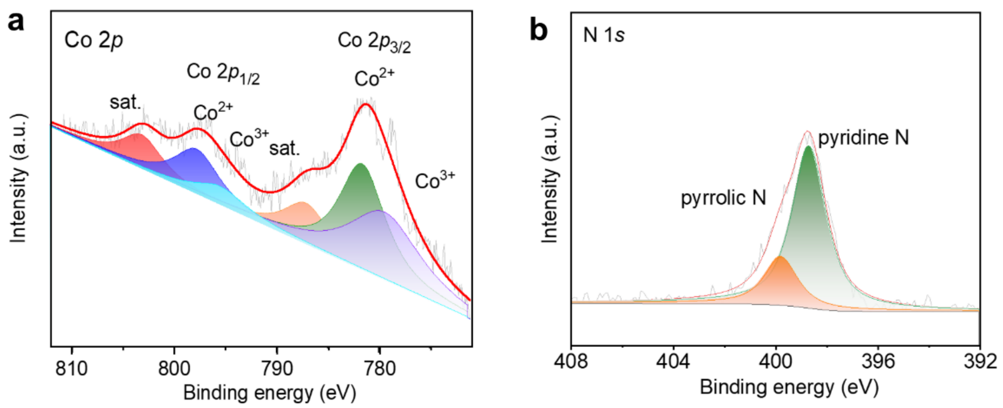

Supplementary Figure 2. Structural characteristic of CN@NiCoS catalyst. High-resolution of XPS spectra (a) Co 2*p*. (b) N 1*s*.

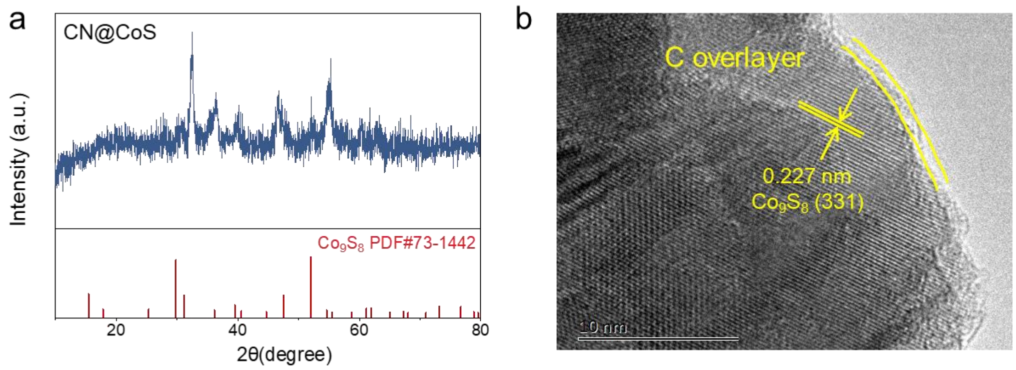

Supplementary Figure 3. Structural characterization of CN@CoS electrocatalyst. (a) XRD profile. (b) HRTEM image.

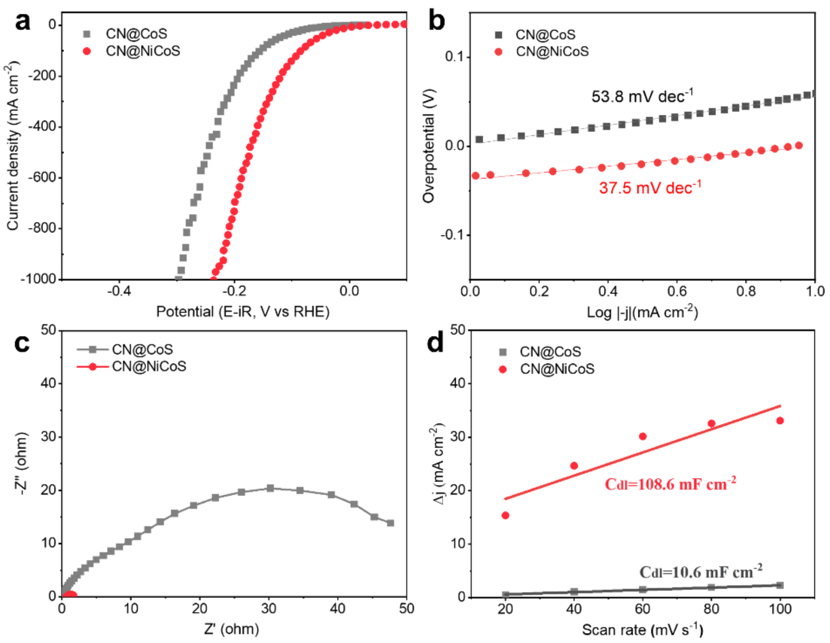

Supplementary Figure 4. Electrochemical performance of CN@CoS and CN@NiCoS electrocatalyst in 1 M KOH solution. (a) LSV curves with iR-corrected. (b) Tafel plots. (c) EIS plots of CN@NiCoS (Rct=2.2Ω) and CN@CoS (Rct=46.6Ω). (d) Calculated electrochemical double-layer capacitance by CV curves with different rates from 20 to 100 mV s^{-1}.

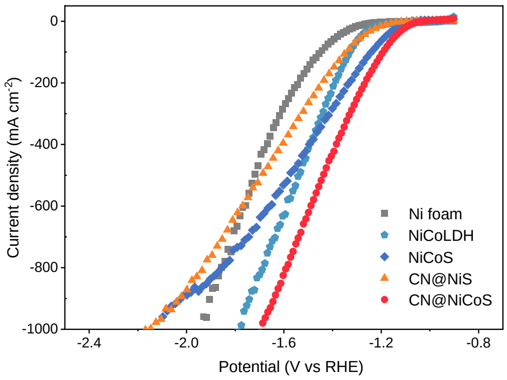

Supplementary Figure 5. Electrochemical performance of different catalysts in alkaline water. LSV curves without iR-corrected of Ni foam (gray), NiCoLDH/NF (green), NiCoS/NF (blue), CN@NiS/NF (orange) and CN@NiCoS/NF (red) electrodes in 1 M KOH solution.

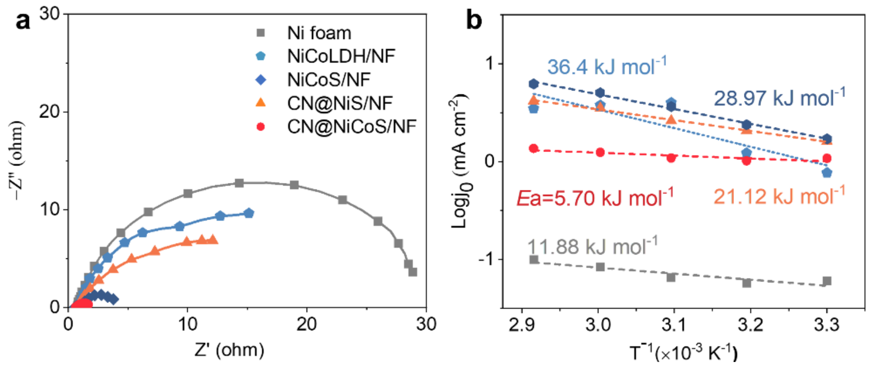

Supplementary Figure 6. Electrochemical performance of different catalyst in 1 M KOH solution. (a) Nyquist plots of Ni foam (*R*ct=29.9 Ω), NiCoLDH (*R*ct=24.2 Ω), CN@NiS(*R*ct=20.4 Ω), NiCoS (*R*ct=3.3 Ω), CN@NiCoS (*R*ct=2.2 Ω). (b) the activation energy (*E*a) of different catalysts. The *E*a was calculated by plots of exchange current density against the inverse of temperature according to Arrhenius equation.

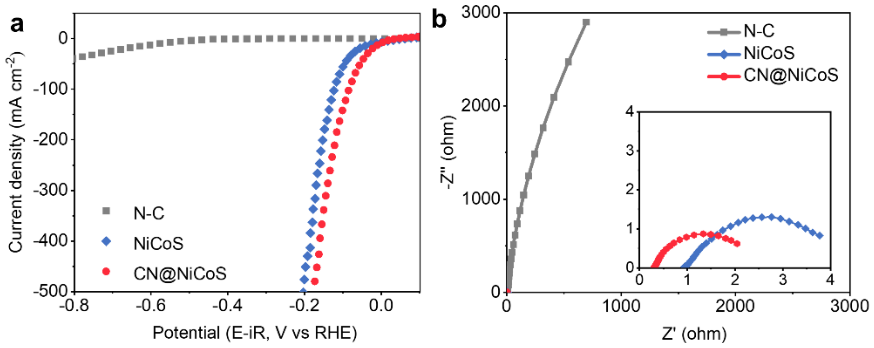

Supplementary Figure 7. Electrochemical performance of N-C, NiCoS and CN@NiCoS. (a) LSV curves with iR-corrected. (b) EIS plots of N-C, NiCoS (*R*ct=3.3Ω), CN@NiCoS (*R*ct=2.2Ω).

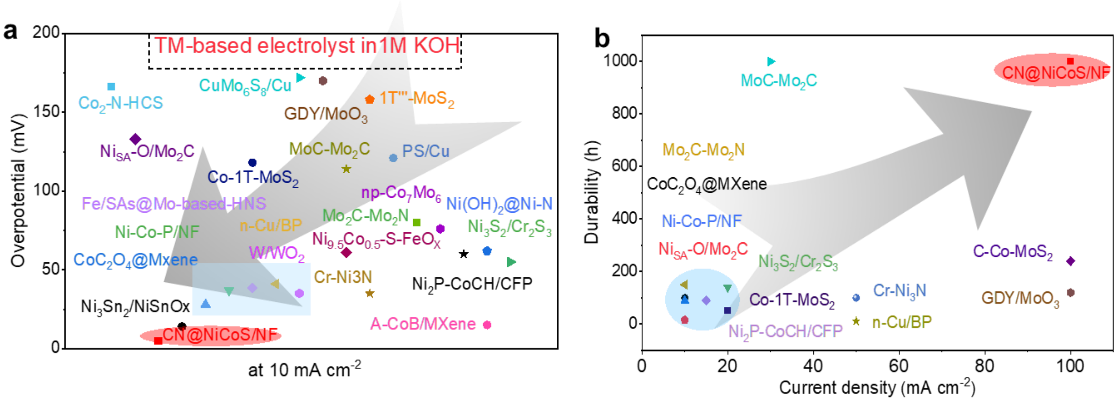

Supplementary Figure 8. Comparison of the activity and stability with reported values of previous HER catalysts in literatures. (a) The overpotentials of transition metal (TM)-based electrocatalysts at current density of 10 mA cm^{-2} in 1M KOH solution. (b) The durability at different current density.

Supplementary Table 1. Comparison of the activity of various electrocatalysts for alkaline HER at room temperature.

Catalysts	Overpotential (mV)	Current density (mA cm^{-2})	Electrolyte	Reference
CN@NiCoS	4.6	−10	1 M KOH	This work
Ni_3S_2/Cr_2S_3	55	−10	1 M KOH	[24]
Ni-Co-P/NF	37	−10	1 M KOH	[26]
Ni_2P-CoCH/CFP	60	−10	1 M KOH	[27]
Ni_3Sn_2/NiSnOx	14	−10	1 M KOH	[28]
$Ni(OH)_2$@Ni-N/Ni-C/NF	60	−10	1 M KOH	[29]
Mo_2C-Mo_2N	80	−10	1 M KOH	[30]
CoC_2O_4@Mxene	28	−10	1 M KOH	[31]
np-Co_7Mo_6	76	−10	1 M KOH	[32]
Fe/SAs@Mo-based-HNSs	38.5	−10	1 M KOH	[33]
PS-Cu	121	−10	1 M KOH	[34]
1T``-MoS_2	158	−10	1 M KOH	[35]
n-Cu/BP	41	−10	1 M KOH	[36]
GDY-MoO_3	170	−10	1 M KOH	[37]
MoC-Mo_2C	114	−10	1 M KOH	[38]
Ni_{SA}-O/Mo_2C	133	−10	1 M KOH	[42]
Cr-NiN/GF	35	−10	1 M KOH	[43]
Co-1T-MoS_2	118	−10	1 M KOH	[44]
Co_2-N-HCS	166	−10	1 M KOH	[46]
W/WO_2	35	−10	1 M KOH	[47]
A-CoB/Mxene	15	−10	1 M KOH	[48]
$Ni_{9.5}Co_{0.5}$-S-FeOx	61	−10	1 M KOH	[49]

Supplementary Table 2. Comparison of the stability of various electrocatalysts for alkaline HER at room temperature.

Catalysts	Stability time (h)	Current density (mA cm^{-2})	Electrolyte	Reference
CN@NiCoS	1000	−100	1 M KOH	This work
Ni_3S_2/Cr_2S_3	140	−20	1 M KOH	[24]
Ni-Co-P/NF	90	−10	1 M KOH	[26]
Ni_2P-CoCH/CFP	90	−15	1 M KOH	[27]
Mo_2C-Mo_2N	150	−10	1 M KOH	[30]
CoC_2O_4@Mxene	100	−10	1 M KOH	[31]
n-Cu/BP	11	−50	1 M KOH	[36]
GDY-MoO_3	120	−100	1 M KOH	[37]
MoC-Mo_2C	1000	−30	1 M KOH	[38]
Ni_{SA}-O/Mo_2C	16	−10	1 M KOH	[42]
Cr-Ni_3N/GF	100	−50	1 M KOH	[43]
Co-1T-MoS_2	50	−20	1 M KOH	[44]
C-Co-MoS_2	240	−100	1 M KOH	[45]

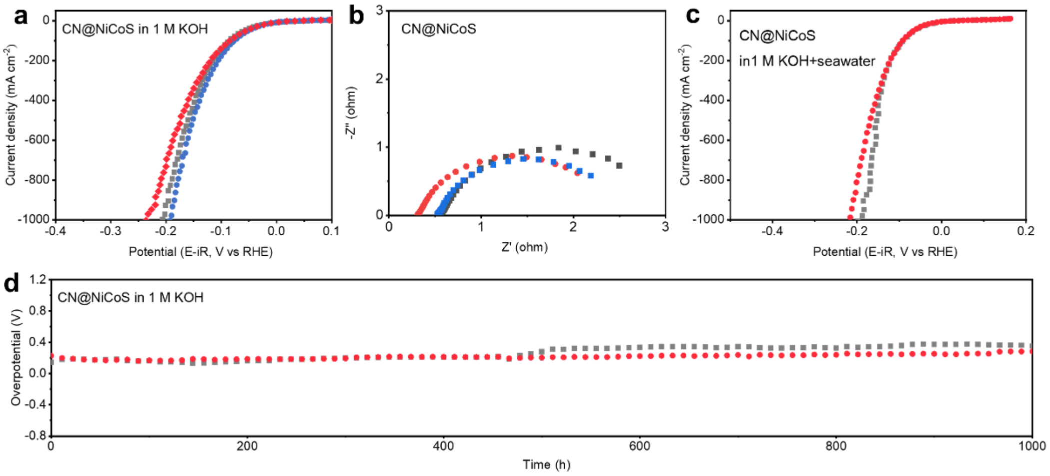

Supplementary Figure 9. **Reproducibility data of CN@NiCoS catalyst**. (a) LSV curves in 1 M KOH solution. (b) EIS plots. (c) LSV curves in 1 M KOH +seawater solution. (d) Durability test in 1 M KOH solution over 1000 h.

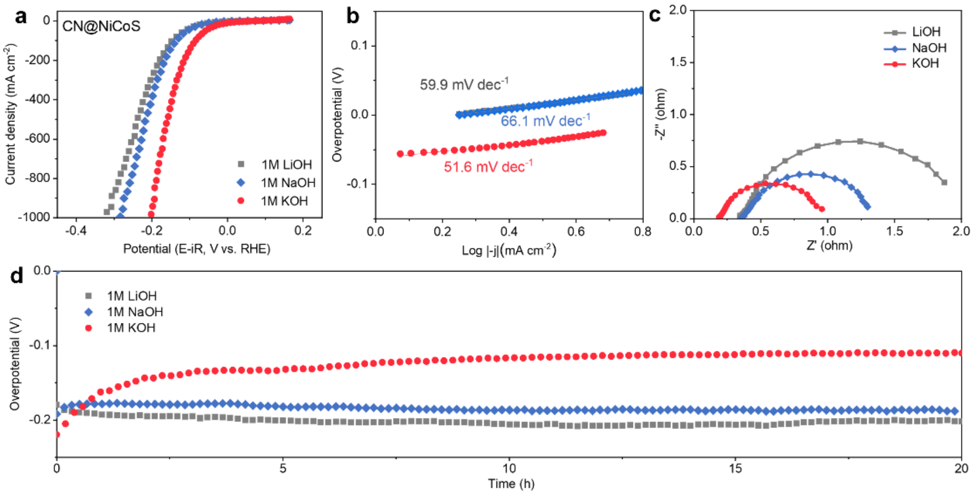

Supplementary Figure 10. Electrochemical performance of CN@NiCoS at pH 14 in different electrolytes with 1 M LiOH, 1 M NaOH and 1 M KOH. (a) LSV curves with iR-corrected. (b) Tafel plots. (c) EIS plots of LiOH (Rct=1.7 Ω), NaOH (Rct=1.0 Ω) and KOH (Rct=0.8 Ω). (d) The chronoamperometry curves of 100 mA cm^{-2} current density under 20 h stability tests.

Note: Regarding the effect of solvated environment, we have investigated the impact of different alkali cations (MOH, M^{+}= Li^{+}, Na^{+} and K^{+}) on the HER performance of CN@NiCoS, while keeping a constant pH value around14. Supplementary Fig. 8a illustrates that the catalytic activity for HER follows the order K^{+}> Na^{+}> Li^{+} across the entire potential range, being consistent with previous reports. Tafel slopes (Supplementary Fig. 8b) and *Rct* values (Supplementary Fig. 8c) decrease in the reverse order Li^{+}>Na^{+}> K^{+}, indicating that an increasing HER kinetic process and fast charge/ion transfer rate in KOH solution. This enhancement can be attributed to a higher concentration of weakly hydrated K^{+} cation near electrode surface, altering the transport rate of cation during the HER process that further could reduce the energy barrier of water adsorption/dissociation. Additionally, the CN@NiCoS catalyst also shows the best excellent long-term stability in KOH electrolyte (Supplementary Fig. 8d). This observation further demonstrates the fast charge/ion transport rate and efficient H_2 evolution capabilities of the CN@NiCoS catalyst in KOH solution.

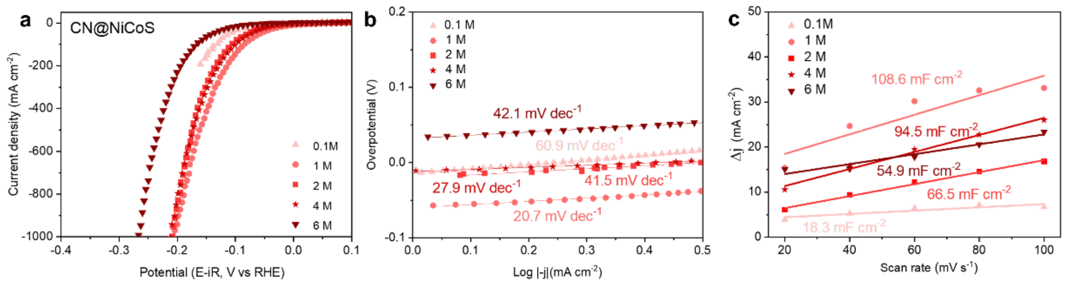

Supplementary Figure 11. Electrochemical performance of CN@NiCoS at KOH solution with different pH values (pH = 0.1 M, 1 M, 2 M, 4 M and 6 M). (a) LSV curves with iR-corrected. (b) Tafel plots. (c) Calculated electrochemical double-layer capacitance by CV curves with different rates from 20 to 100 mV s^{-1}.

Note: To elucidate the pH effect, we further examined the electrochemical performance of CN@NiCoS with different pH values under alkaline KOH conditions. As shown in Supplementary Fig. 9, we observed an enhanced HER activity with increasing pH from 0.1 M to 1 M, which dramatically drops when further increasing the pH to 6M. This can be attributed to the improved electrical conductivity due to the increased ion concentration at low pH, whereas the hydrogen binding energy (HBE) plays a dominant role in higher pH alkaline media that further impede the HER activity.

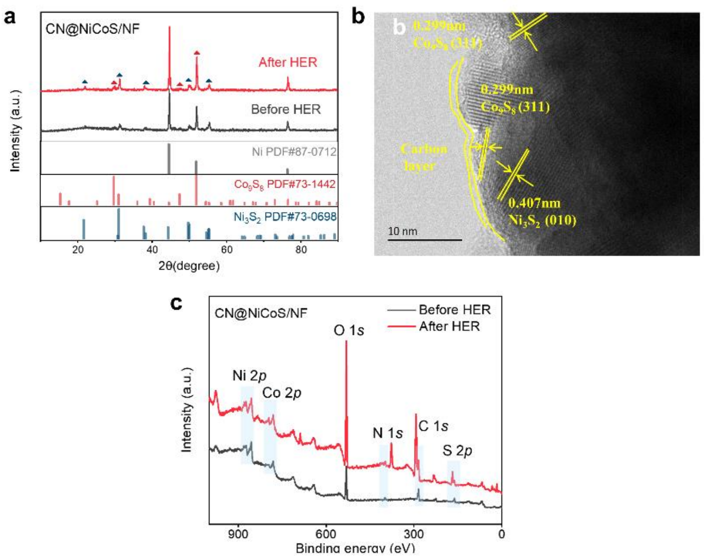

Supplementary Figure 12. The morphology and structure characteristic of CN@NiCoS before and after HER. (a) XRD patterns. (b) HRTEM images. (c) XPS survey spectrum.

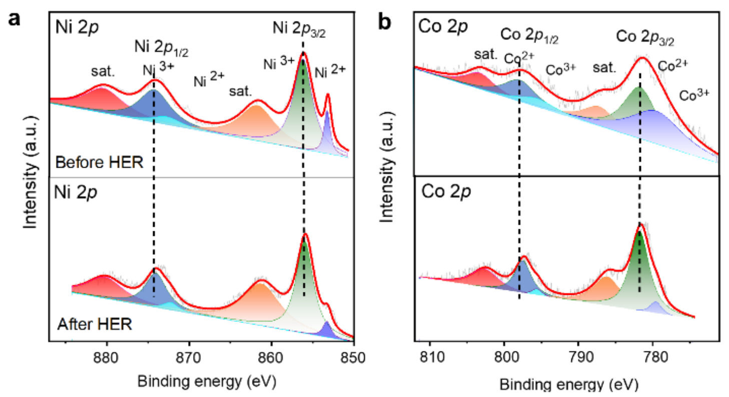

Supplementary Figure 13. Structural characteristic of CN@NiCoS before and after HER.

High-resolution of XPS spectra (a) Ni 2*p*. (b) Co 2*p*.

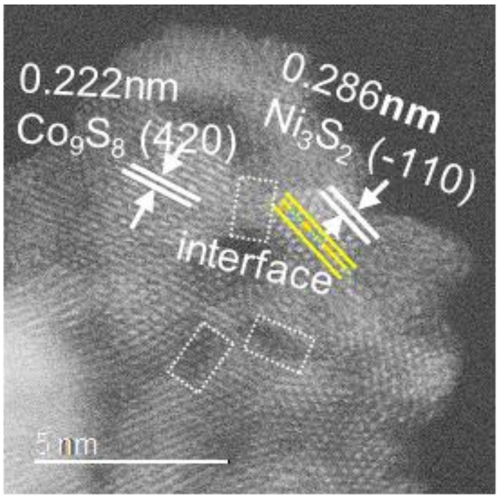

Supplementary Figure 14. Morphology characteristic of CN@NiCoS catalyst. HAADF-STEM images of CN@NiCoS after HER stability test.

Supplementary Table 3. Sulfur concentration in electrolyte after 100 h HER. Inductively coupled plasma optical emission spectrometry (ICP-OES) data of electrolyte stoichiometry after NiCoS, C@NiCoS and CN@NiCoS HER testing.

	S concentration in electrolyte (mg/L)
NiCoS	2.406
C@NiCoS	0.379
CN@NiCoS	0.133

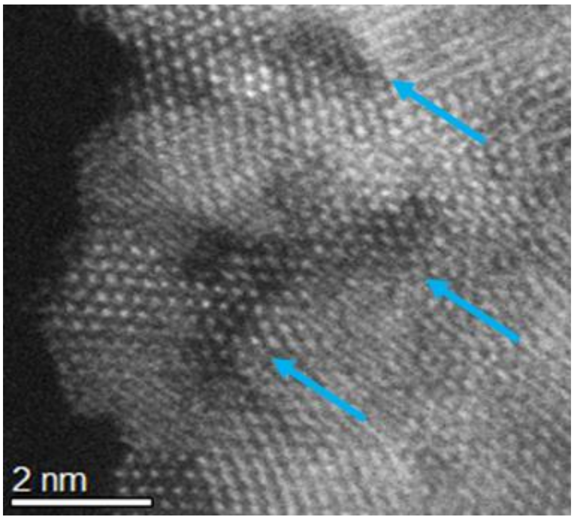

Supplementary Figure 15. Morphology characteristic of NiCoS catalyst. HAADF-STEM images of NiCoS catalyst after HER.

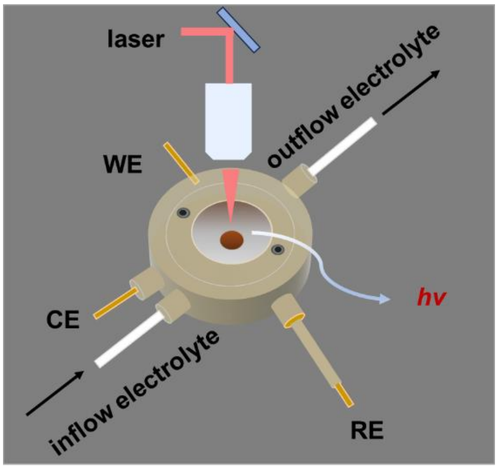

Supplementary Figure 16. Schematic of in situ Raman electrochemical cell. Pt electrode, Ag/AgCl and different electrode were used of counter electrode (CE), reference electrode (RE) and working electrode (WE), respectively.

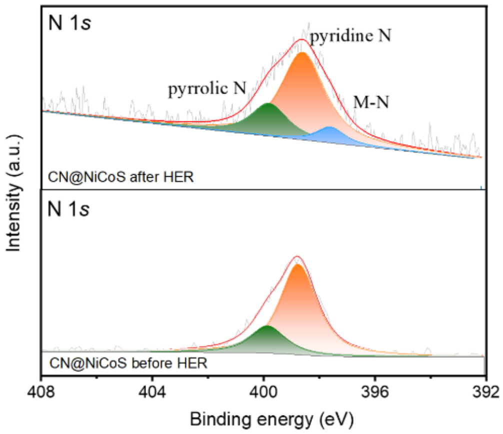

Supplementary Figure 17. Surface phase composition of CN@NiCoS catalyst. XPS spectra for N 1*s* before and after HER.

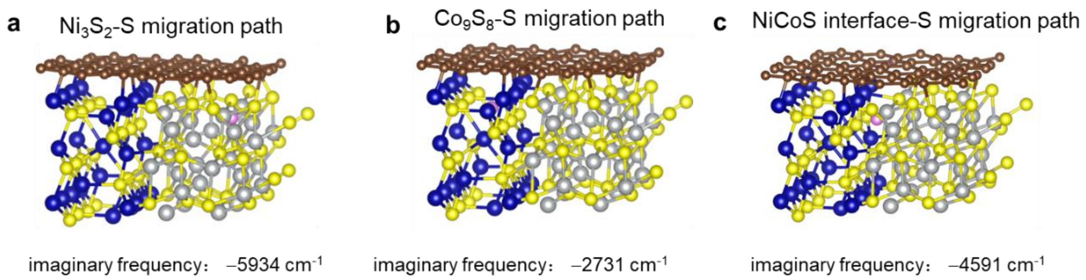

Supplementary Figure 18. DFT calculation of sulfur migration path at different sulfides. The transition state structure and corresponding imaginary frequency during sulfur migration process (a) Ni_3S_2 phase. (b) Co_9S_8 phase. (c) NiCoS heterojunction phase.

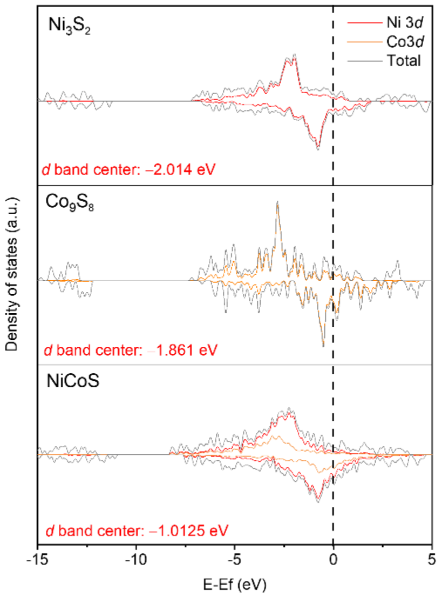

Supplementary Figure 19. DFT calculation in the role of heterojunction. The density of states (DOS) of Ni_3S_2, Co_9S_8 and NiCoS.

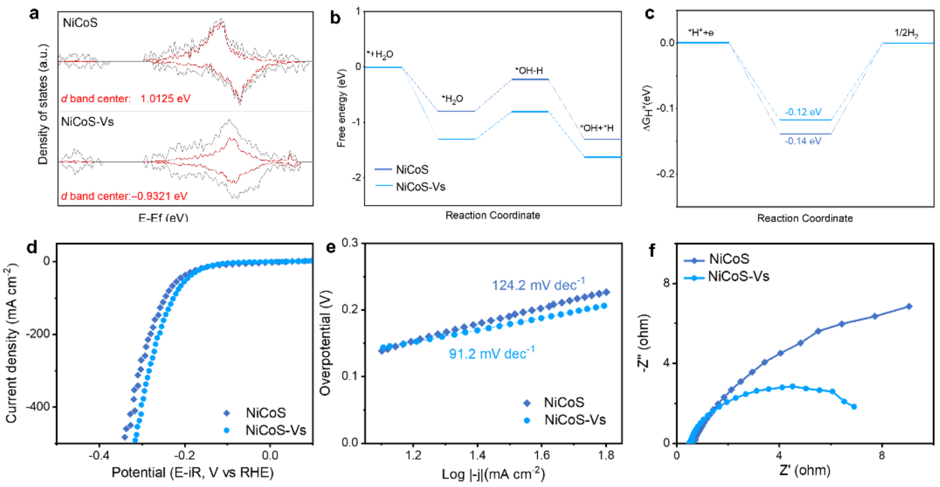

Supplementary Figure 20. Density Functional Theory (DFT) calculations and electrochemical performance of NiCoS and NiCoS-Vs. (a) The density of states (DOS). (b) Water dissociation energy barrier. (c) Hydrogen evolution energy barrier. (d) LSV curves with iR-corrected. (e) Tafel plots. (f) Nyquist plots.

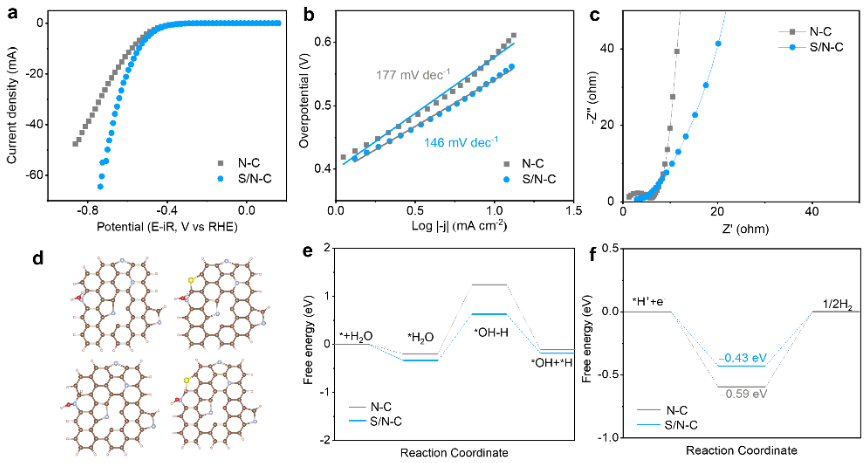

Supplementary Figure 21. Electrocatalytic HER performance and DFT calculation of N-C and S/N-C. (a) Linear sweep polarization curves. (b) Tafel slopes. (c) Nyquist plots. (d) Density Functional Theory model of N-C and S/N-C. Reaction free-energy diagram of HER on N-C and S/N-C. (e) Water dissociation. (f) Hydrogen evolution.

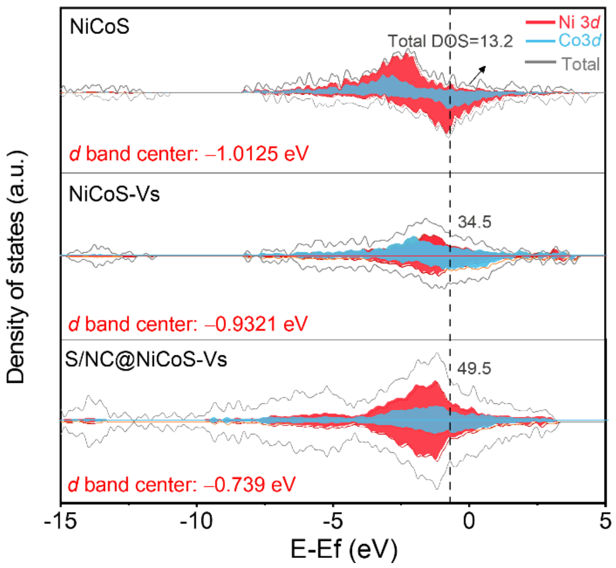

Supplementary Figure 22. DFT calculation for the hybridization of Ni 3*d* and Co 3*d* orbital.

The density of states (DOS) of NiCoS, NiCoS-Vs and S/NC@NiCoS-Vs.

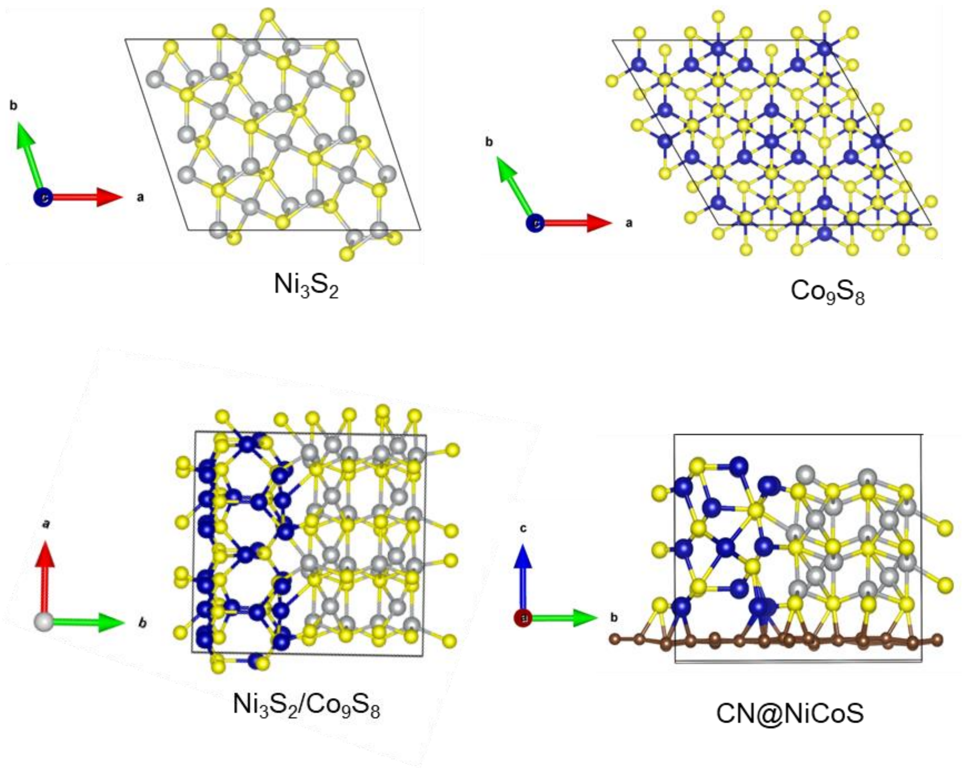

Supplementary Figure 23. Optimized structural models. Computational models of different catalyst (a) Ni_3S_2. (b) Co_9S_8. (c) Ni_3S_2/Co_9S_8. (d) CN@NiCoS. Yellow, S; Gray, Ni; Blue, Co; Brown, C.

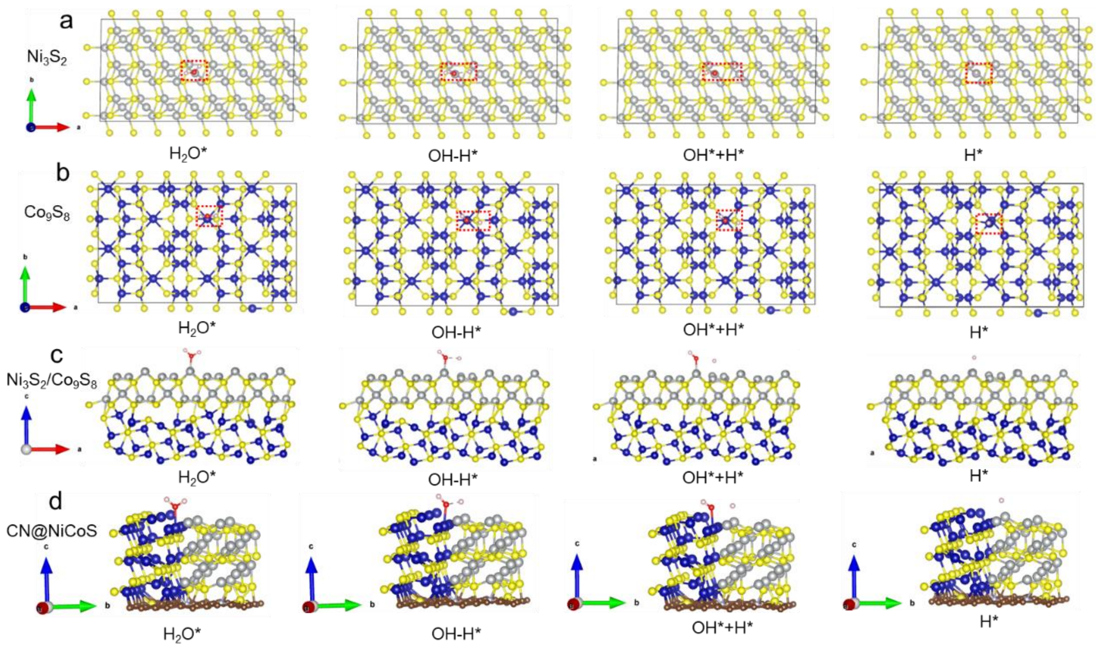

Supplementary Figure 24. Optimized atomic configuration for different catalyst of hydrogen-containing species (H_2O*, OH-H*, OH*+H* and H*). (a) Ni_3S_2. (b) Co_9S_8. (c) Ni_3S_2/Co_9S_8. (d) CN@NiCoS.

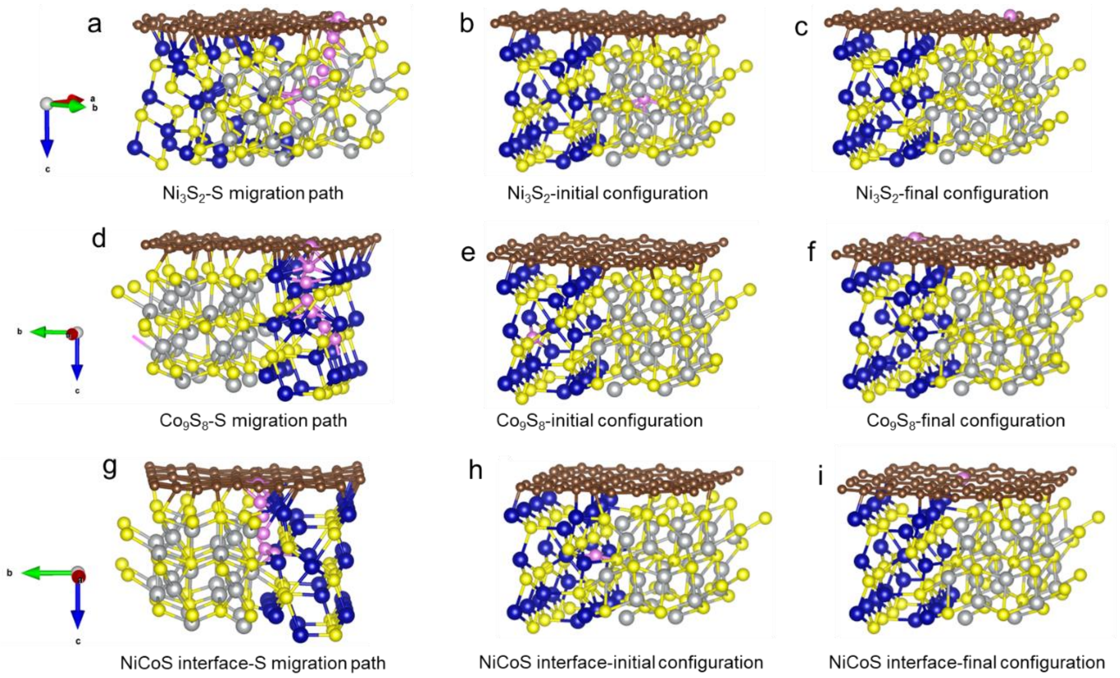

Supplementary Figure 25. Sulfur migration trajectory in Ni_3S_2, Co_9S_8 and NiCoS phase with the initial and final configurations. (a-c) S migration path in Ni_3S_2 phase and initial/final configurations. (d-f) S migration path in Co_9S_8 phase and initial/final configurations. (g-i) S migration path in NiCoS phase and initial/final configurations.

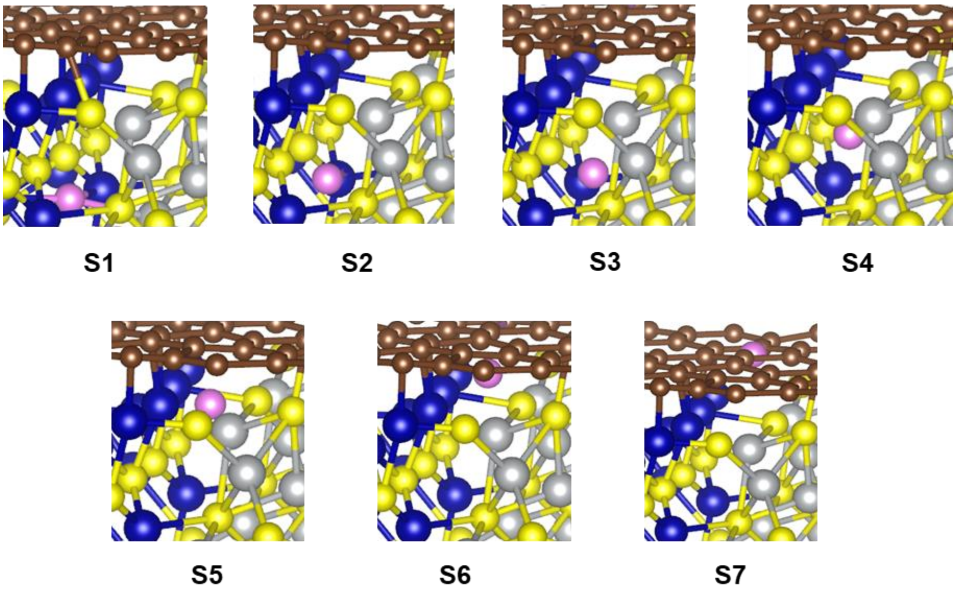

Supplementary Figure 26. DFT structural model of sulfur migration. S migration trajectory from initial S1 to final states S7 in NiCoS interface.
